# Hemiparkinsonism or Hemidystonia With Hemiatrophy Syndrome: A Case Series With Follow-Up

**DOI:** 10.3389/fnins.2020.00064

**Published:** 2020-02-04

**Authors:** Chentao He, Piao Zhang, Yan Li, Bing Li, Zhiheng Huang, Lijuan Wang, Yuhu Zhang

**Affiliations:** Department of Neurology, Guangdong Neuroscience Institute, Guangdong Provincial People’s Hospital, Guangdong Academy of Medical Sciences, Guangzhou, China

**Keywords:** hemiparkinsonism, hemidystonia, hemiatrophy, PET, follow-up, heterogeneity

## Abstract

Hemiparkinsonism-hemiatrophy syndrome (HPHA) and hemidystonia-hemiatrophy syndrome (HDHA) are rare movement disorders composed of hemidystonia or hemiparkinsonism that present with unilateral limb, face, trunk, or cerebral hemiatrophy and mostly occur following head trauma or postanoxic events. However, relatively little is known about the pathogenesis of these conditions. In our case series, we present three HPHA patients and one HDHA patient who underwent detailed neuropsychological, radiological, motor, and non-motor functional assessments with a mean follow-up of 2 years. We followed two patients who showed differences in their progression for more than 2 years: one barely progressed with no treatment, and the other exhibited levodopa-induce dyskinesia (LID) and definitive progression while receiving multiple adjunctive therapies. In addition, we performed positron emission tomography (PET) with 18F-fluorodeoxyglucose (FDG) and 18F-dihydroxyphenylalanine (DOPA) in one HPHA patient who showed bilaterally symmetrical uptake of FDG with no significant increase or decrease in the cerebral hemispheres, including the striatum, but exhibited a significant reduction in the uptake of 18F-DOPA in the contralateral posterior striatum. In this study, we followed HPHA patients who showed different disease courses to explore the clinical characteristics and pathogenesis of HPHA and HDHA and illustrate the clinical heterogeneity of these diseases.

## Introduction

Hemiparkinsonism-hemiatrophy syndrome (HPHA) is a rare form of secondary parkinsonism. Its clinical manifestations include the occurrence of unilateral atrophy on one side of the body, which often presents early in life, and ipsilateral hemiparkinsonism with different latencies and relatively slow progression. HPHA is frequently accompanied by dystonia on the same side and shows a variable response to levodopa. Hemidystonia-hemiatrophy syndrome (HD-HA) is an uncommon condition characterized by hemidystonia and hemiatrophy. In one study, hemiparesis preceded HD in 88% of HDHA cases (Wijemanne and Jankovic, 2009). Dystocia, premature delivery, perinatal hypoxia, and fever during infancy or head trauma in early childhood all seem to play a key role in its underlying pathogenesis. Here, we described a series of HPHA cases with follow-up in an attempt to further explore the clinical characteristics and pathogenesis of the spectrum of these disorders, further illustrating the clinical heterogeneity of this disease.

## Case 1

**FIGURE 1:**
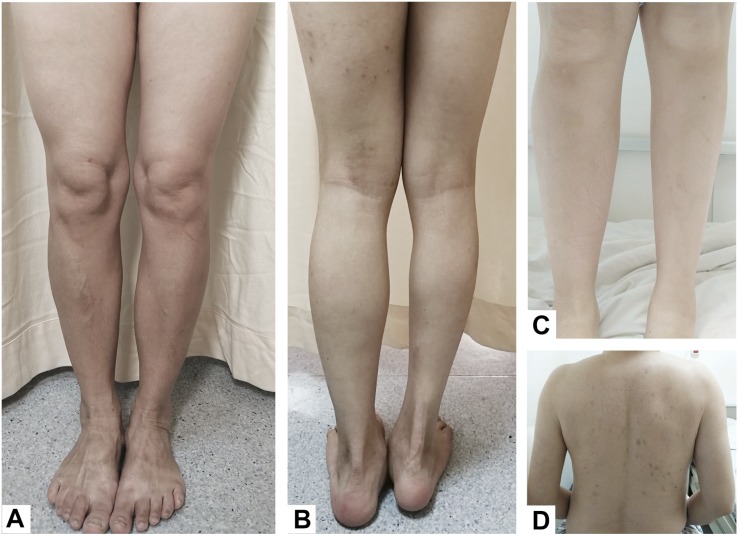
**(A)** Case 3, hemiatrophy of the left-sided lower limb. **(B)** Case 4, the right lower limb was significantly shorter and smaller than the left lower limb, and the right foot was in an abducted posture. **(C)** Case 1, compared to the right calf, the left calf showed hemiatrophy. **(D)** Case 2, atrophy of the left arm and back muscles.

A 28-year-old, right-handed female with hemiatrophy in her left-sided extremities and face that became apparent when she was 2 years old presented with clumsiness in her left lower limb while climbing stairs and an abnormal dragging gait that appeared gradually. The patient was otherwise healthy until she was 28 years old. At this age she began to feel weakness and slowness in her left lower limb, which gradually worsened. These symptoms were relieved after taking a break, even though she had not used any drugs over the years. She was born via cesarean section due to a lack of amniotic fluid, which resulted in a suspected history of perinatal hypoxia. There was a negative family history for any neurological disorder and no history of brain injury or exposure to toxins and medications associated with parkinsonism. On examination, the left side of her face, tongue, and limbs were smaller than those on the right side (Figure 1C). Mild rigidity was apparent in the left upper and lower limbs. She had an abnormal posture that made her leg appear bow-shaped toward the left foot, and her dorsiflexion was slightly restricted. No tremor was observed, and no abnormal clinical manifestations were observed in the right extremities. The deep tendon reflex was brisker in the left than the right lower limb. There were positive bilateral Rossolimo signs and left Babinski signs, but the right Babinski signs were suspect. Muscle strength, the cranial nerves and sensation, and mental and cognitive functions were normal. Brain and cervical spine magnetic resonance imaging (MRI) and calf X-rays were unremarkable. The lateral ventricles were symmetrical, and no abnormalities were detected in subcortical structures. Bilateral surface electromyography (EMG) was normal. The Hoehn-Yahr stage was 1.5. The levodopa response was poor [Unified Parkinson’s Disease Rating Scale-Part III (UPDRS-III) motor scores for both on-medication and off-medication were 9] after the first levodopa dose in the morning. The patient felt no subjective improvement after a 200-mg dose of levodopa. Medical exome sequencing was negative and showed no mutations. We followed the patient, and, 3 years later, her symptoms were stable despite not taking any medicines during that time, and she denied any progression in left-side disorders or any abnormality on the right side.

**TABLE 1:** Demographic and clinical data.

Clinical features	Case 1 (HPHA)	Case 2 (HPHA)	Case 3 (HPHA)	Case 4 (HDHA)
Sex, side	F, L	M, L	F, L	M, R
Abnormal birth or history of injury	Perinatal hypoxia	No	Head trauma	Head trauma
Age at onset	2	50	48	5
First symptom	Dystonia, dragging gait	Bradykinesia	Rest tremor, dragging gait	Dystonia
Dystonia	Present	Absent	Absent	Present
Response to levodopa	Poor	Moderate	Moderate	Poor
Follow-up, years	3	2	1	−
Progression	No	Aggravation, dyskinesia	No	−
Progressed to bilateral	No	Yes	No	No
Brain MRI 3.0T	Unremarkable	Moderate widening of the ventricles, mild brain atrophy	Unremarkable	Dilatation of the left lateral ventricle
18F−FDOPA PET	−	−	Significant reduction in the uptake of 18F−DOPA in the right posterior striatum	−
18F−FDG PET	−	−	Normal	−
Medical exome sequencing	Normal	Normal	Normal	Normal

## Case 2

**FIGURE 2:**
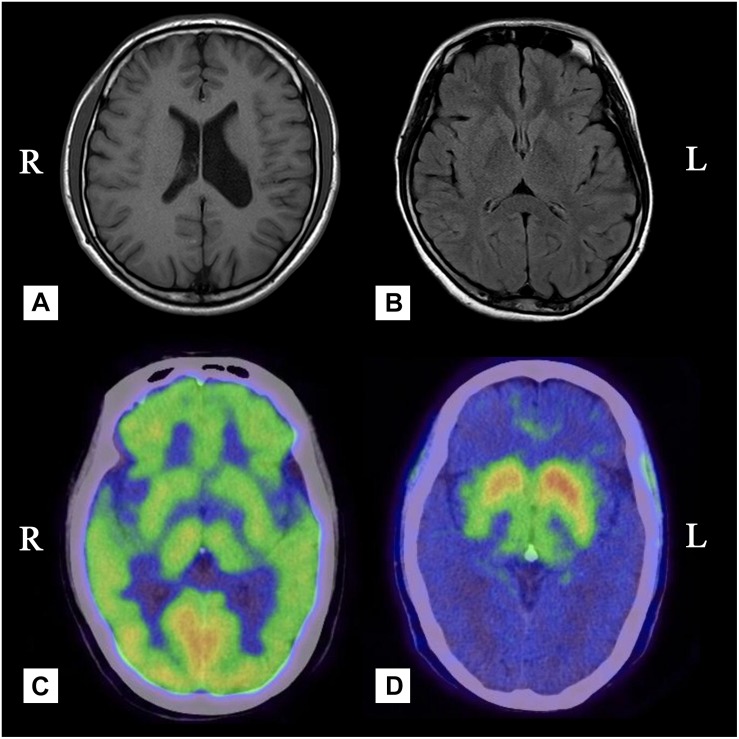
**(A)** Case 4, Brain MRI showing dilatation of the left lateral ventricle. **(B)** Case 2, Brain MRI showing mild brain atrophy but no signs of hemisphere hypoplasia. **(C)** Case 3, PET with [18F]-FDG was bilaterally uniform and symmetrical. **(D)** Case 3, there was a significant reduction in the uptake of 18F-DOPA in the contralateral posterior striatum.

A 55-year-old, right-handed male who was born via spontaneous vaginal delivery at term without dystocia complained of a slowness of the left arm and leg on walking that started at 50 years of age. At the same time, he also became aware of atrophy in his left-side limbs and back muscles (Figure 1D). A series of neurological examinations performed when he was aged 55 years showed mild cogwheel rigidity, bradykinesia, and a reduction in his arm swing on walking along with same-side hemiatrophy. Muscle strength was level 4 in the left limbs. The deep tendon reflexes were brisk on both sides, and the pathological sign was negative. The Modified Hoehn and Yahr scale was 1.5. His UPDRS-III motor score was 13/108 before and 9/108 after the first morning levodopa dose (slowing of finger tapping and hand movements on the left and left limb rigidity but normal on the right). Additionally, there were no abnormalities in an electroencephalogram examination of somatosensory-evoked potentials, event-related potentials, auditory-evoked potentials, brainstem auditory-evoked potentials, motor-evoked potentials, and visual-evoked potentials nor in the transcranial doppler sonography (TCD), olfactory function tests, urinary flow rate, and bladder function. Cognition and cranial nerve function were both intact. The Self-Rating Anxiety Scale (SAS) and the Self-Rating Depression Scale (SDS) showed mild depression. Brain MRI 3.0T showed moderate widening of the ventricles and mild brain atrophy but no signs of hemisphere hypoplasia (Figure 2B). Levodopa (325 mg/d) and ropinirole (4 mg/d) were administered and provided mild improvement in the 1st year. In the 2nd year, dyskinesia and end-of-dose deterioration were present on the affected side, but there was no dystonia. Benzhexol (2 mg/d) was added and extended the on-period to 1.5 h. Then, in the 3rd year, the addition of amantadine (200 mg/d) improved his parkinsonism and dyskinesia signs. At that time, his UPDRS-III motor score (in the morning without antiparkinsonian therapy) was 24, and he showed some deterioration compared with 2 years prior. Bradykinesia and rigidity began to appear on the right extremities, although the left extremities remained the major affected extremities.

## Case 3

**FIGURE 3:**
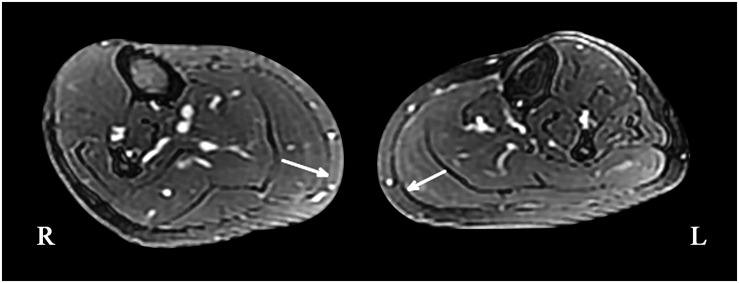
Magnetic resonance imaging of the bilateral calf muscles; asymmetry of the posterior muscles of the left calf can be seen on cross-section (maximum diameter: right side, 21.53 mm; left side, 17.03 mm).

A 49-year-old, right-handed woman presented with left-side hemiatrophy and resting tremor. She had been aware of a left-side dragging gait while walking since she was 48 years old, and this was followed by slowness on walking 2 months later. At the same time, resting tremors appeared in the left upper limb, and a clinician observed that her body showed hemiatrophy (Figure 1A). Her right-side limbs were normal. There was no relevant family history, traumatic birth, or significant childhood illness. She had fallen from two meters when she was 7 years old, but reported no noticeable discomfort. On physical examination, there was prominent exophthalmos in both eyes. The left limbs showed mild rigidity when completely relaxed, and this was not observed on the right side. There was a reduction in her left upper limb swing while walking. The resting tremor was prominent at approximately 4 to 6 cycles per second (Hz) in her right hand. Other neurological examinations, such as strength, autonomic function, and sensation, were unremarkable. There were no autonomic dysfunctions, such as nocturia, constipation, or orthostatic hypotension. Her UPDRS-III motor score was 16 before and 14 after the first morning levodopa dose, after which the rigidity in her left upper limb improved significantly. In addition, we used a series of scales to assess cognitive function, including the Mini-Mental State Examination (MMSE) and the Montreal Cognitive Assessment Beijing Version, and found no abnormalities in these assessments. However, the Hamilton Anxiety Scale showed suspected anxiety, and the Hamilton Depression Scale (24-item version) score was 25, indicating she had mild depressive symptoms. We performed MRI of the bilateral calf muscles. Asymmetry of the posterior muscles of the calves was observed on cross-section (Figure 3); steatosis, edematous, necrosis, and inflammation were not found. ^18^F-Dihydroxyphenylalanine Positron Emission Tomography (18F-DOPA PET) showed a significant reduction in the uptake of 18F-DOPA in the right posterior striatum (Figures 2C,D), while ^18^F-fluorodeoxyglucose (FDG) uptake was bilaterally uniform and symmetrical. No genetic abnormalities were detected by medical exome sequencing.

## Case 4

A 24-year-old man with a normal perinatal history had fallen from the second floor and struck the left side of his head without loss of consciousness. Since then, atrophy of his right limbs and trunk was gradually noticed, and this was followed by dystonia and motor impairment on his right side. On examination at an age of 24 years, his right limbs were significantly shorter and smaller than his left limbs (Figure 1B), and he had an abnormal posture (limited right hand movements, abducted right foot when resting). No obvious abnormalities were found on the left side. Mild rigidity was detected in the right limbs but not on the left side. There was slight slowness and limited hand movements on the right as well as reduced amplitudes for right finger and toe tapping, although these symptoms were difficult to attribute to either parkinsonism or dystonia. His right lower limb internally rotated as he walked. The Rossolimo sign of the right hand and the Chaddock sign of the right foot were positive. An MRI scan showed dilatation of the left lateral ventricle (Figure 2A). EMG did not show lower motor nerve damage, consistent with his clinical symptoms. We performed next-generation medical exome sequencing on this patient and his parents, but no mutations associated with these clinical manifestations were detected. In addition, he did not respond to a 200-mg dose of levodopa.

## Discussion

Hemiparkinsonism-hemiatrophy syndrome (HPHA) is a relatively rare type of secondary parkinsonism. The clinical manifestations of HPHA are heterogeneous. In the relevant HPHA-related literature, hemiatrophy (including of the unilateral limbs, face, or trunk) and hemihypertrophy often occur early in life, and parkinsonism onset occurs in subsequent years or decades. The interval from HA to HP was variable. The average age of onset of parkinsonism was 43.7 years in a study of 16 HPHA patients (Buchman et al., 1988). Parkinsonian symptoms, including bradykinesia, dystonia, or rest tremor, and some other symptoms were reported. A previous study reported a case with unusual exertion-induced weakness and hypotonia alternating with hypertonia and dystonia (Lang, 1995). Eleven of the 30 (37%) patients in another study had scoliosis (Wijemanne and Jankovic, 2007). Some related studies have shown that parkinsonism generally appears earlier than idiopathic Parkinson’s disease (PD) and progresses slowly in most cases. This may be because of its distinct association with neuroplasticity, dopamine reserve, and compensation mechanisms, which depend, to varying extents, on cerebral lesions. Some cases have also been reported that developed bilateral symptoms after varying numbers of years. However, the symptoms in these cases still mainly occurred on the initially affected side. During the follow-up period, the symptoms in two early-onset patients had not progressed to the contralateral side. In contrast, while case 2 developed bilateral symptoms and exhibited dyskinesia in the following 2 years, the patient’s symptoms mainly occurred on the left side.

Gowers described the relationship between hemispheric lesions and contralateral limb atrophy, which the author attributed to a head injury followed by cortical abnormalities (Gowers, 1878). Penfield suggested that hemiatrophy represented a dysplasia and not a true “atrophy” and that contralateral body atrophy most likely resulted from postcentral lesions that occurred before patients were 3 years old (Penfield and Robertson, 1943). A variety of lesions have been shown to be related to hemiatrophy; these include lesions of the postcentral gyrus, cortex, subcortical white matter, mesencephalon, and basal ganglia, in particular the putamen (Greene et al., 2000). In contrast, ipsilateral bone atrophy may be attributed to cortical lesions (Wijemanne and Jankovic, 2009). The asymmetry of the body and other symptoms of the patient may be related to the initial differences in the cells that were lost in the bilateral cerebral hemispheres (Giladi et al., 1990). However, the specific relationship between HP and HA remains unclear. Dystocia, premature delivery, perinatal hypoxia, and fever during infancy or head trauma in early childhood probably account for the majority of these syndromes. In one study, nearly half of the patients (47%) experienced cerebral hypoxia during birth or infancy (Wijemanne and Jankovic, 2007). Similarly, three patients in our study had suspected delivery complications or injuries in early life.

Eighty-eight percent of HDHA patients who present with hemiparesis preceding HD, which appeared after a mean 14.7-year latency. Patients who were younger at the onset of cerebral insult tended to have a later onset of dystonia, regardless of the cause of injury (Wijemanne and Jankovic, 2009). However, unlike previous HDHA reports, in our study, case 4 presented with hemiparesis and dystonia almost simultaneously. He was injured at 5 years old and showed no further progression until now. Aside from difficulties with rapid movements of the right limbs, he showed no signs of parkinsonism. We diagnosed his disease as HD-HA but not HPHA. Many studies have demonstrated that there is potentially mechanistic overlap between dystonia and parkinsonism. Dystonia, in which sustained or intermittent muscle contractions cause abnormal postures, is often initiated or worsened by voluntary actions and has been associated with overflow muscle activation (Albanese et al., 2013). Haggstrom argued that mild parkinsonian signs are an additional manifestation of dystonia arising from basal ganglia dysfunction and that movement may also be slow or delayed in patients with dystonia. True decremental bradykinesia may be the most discriminating feature for distinguishing dystonic syndromes from PD (Haggstrom et al., 2017).

With the exception of the brain MRI performed in case 4, which showed dilatation of the left lateral ventricle, the other three patients had normal and symmetrical brain MRI scans. The abnormal findings observed on cranial MRI in patients with HPHA can be widely grouped into four categories: (1) focal atrophy or diffuse cerebral HA, (2) single or multiple focal lesions in the basal ganglia, (3) single or multiple focal lesions outside of the basal ganglia system, which are probably incidental findings unrelated to the underlying pathophysiology, and (4) normal scans (Wijemanne and Jankovic, 2007). Nine (30%) patients in one study showed enlargement of the lateral ventricle on the affected side with a reduction in cortical and subcortical volumes, in contrast to the findings found in idiopathic PD, which is associated with nearly normal imaging results. A total of 33% of the scans were interpreted as normal. In 2016, Barbagallo reported a patient with midbrain hemiatrophy and nigral rarefaction (Barbagallo et al., 2016). Parry-Romberg syndrome, which is characterized by progressive facial hemiatrophy and ophthalmological manifestations, also show ipsilateral cerebral atrophy in brain MRI. However, unlike the HPHA or HDHA, the motor function and the muscles of trunk or extremities are not affected.

Findings observed on FDOPA-PET can reflect the function of presynaptic dopaminergic neurons. One of our HPHA patients showed a significant reduction in the uptake of 18F-DOPA in the right posterior striatum, consistent with previous reports indicating that dysfunction in presynaptic dopaminergic neurons occurs in HPHA patients. FDG-PET reveals the integrity of neurons and synaptic functions in general. FDG uptake increases with synaptic activity and decreases with neural dysfunction. In our case 3 patient, FDG uptake was bilaterally symmetrical, and no significant increase or decrease was observed, including in the striatum. This is different from what has been reported in previous investigations that showed metabolic reductions in the contralateral or bilateral striatum or cortices (Przedborski et al., 1993; Przedborski et al., 1994; Wijemanne and Jankovic, 2007; Yang and Kwak, 2015). Our results suggest that HPHA likely involves compensatory mechanisms that affect the overall neuronal metabolism of the striatum because dopaminergic neurons account for only 10–15% of striatal synapses. In addition, the PD-related pattern (PDRP) is consistently characterized by relatively increased or maintained metabolism in the striatum (Stoessl and Mckeown, 2016; Meles et al., 2017), which is different from the metabolic patterns observed in HPHA; this difference may help physicians distinguish HPHA from Parkinson’s disease. Nonetheless, this was observed in only a single case, and more follow-up studies need to be performed in the future that focus on the mechanisms involved in striatal neuronal metabolism since this information may be helpful for determining the underlying pathogenesis of HPHA. Greene proposed that hemiatrophy may be caused by early asymmetric deficits in dopaminergic transmission (Greene et al., 2000). A reduction in dopamine transporters has been correlated with the loss of dopaminergic neurons in the striatum and may lead to contralateral parkinsonism (Pisani et al., 2011).

Pramstaller presented one HPHA female patient with parkin mutations (Pramstaller et al., 2002). Peker found that the knockdown of the Parkin gene led to myotubular atrophy *in vitro*, an effect that was associated with impaired mitochondrial function and a smaller myofiber area (Peker et al., 2018). Consequently, the genetic etiology of HPHA and HDHA, which include parkin mutations, cannot be fully excluded. To achieve an accurate genetic diagnosis, medical exome sequencing was carried out in our study. In all, 74566 genomic regions spanning 12424088 bp were sequenced, and coverage of 99% of the sequenced regions exceeded 20X in all of the patients. The sequence analysis indicated that no variants, including those in the Parkin gene, were identified as being etiologically responsible for the clinical manifestations observed in the investigated patients. However, our analysis cannot exclude the involvement of the deep intronic regions of potentially related genes, and there may also be novel genetic causes for the phenotypes of the three patients investigated in our study, or genetic factors may not be the cause of the disease in these patients.

Among our four cases, two showed absolutely no response to levodopa, while the other two cases had a slight response to levodopa. Not all patients with HPHA or HDHA exhibit a good response to levodopa. Klawans suggested that the response to levodopa was minimal in most of their patients in their 1981 study (Klawans, 1981), while subsequent studies showed that the majority of patients showed a satisfactory response to levodopa treatment. These differences were most likely related to different extents of striatal dysfunction. However, parkinsonism resulting from nigral disease responds well to levodopa. Ayromlou described a patient who showed a good response to the addition of a dopamine agonist to levodopa therapy (Ayromlou et al., 2011). In addition, surgery, such as deep brain stimulation (DBS), may exert some effect on the associated parkinsonism and dystonia reported in patients in some previous studies.

Hemiparkinsonism-hemiatrophy syndrome and HDHA are both heterogeneous disorders that occur following static cerebral injury during young age or the perinatal period, as was the case in two HPHA patients and one HDHA patient in our report. A variety of lesions are related to hemiatrophy, particularly those that occur in the basal ganglia (Greene et al., 2000; Wijemanne and Jankovic, 2009). Reduced uptake of 18F-DOPA in our study (case 3) implied the dysfunction of presynaptic dopaminergic neurons in the striatum, contributing to contralateral parkinsonism. Therefore, we hypothesized that the cooccurrence of hemiatrophy, contralateral dystonia, and parkinsonism might be attributed to basal ganglia lesions. Because some patients with HPHA also show dystonia, it is reasonable to assume that the pathogenesis underlying both HPHA and HDHA is common in certain aspects. These syndromes may be part of a spectrum of movement disorders.

## Conclusion

In conclusion, in reporting this case series and follow-up in patients with different disease courses, we explored the clinical characteristics and pathogenesis of HPHA and HDHA, further illustrating the clinical heterogeneity of these diseases. Considering the slow progress observed in the majority of patients with these conditions, it is necessary to recognize that hemiatrophy accompanies parkinsonism or dystonia. In addition, an abnormal birth or history of injury may also be a contributing factor. Brian MRI or PET might help to identify the pathogenesis of these conditions. A large prospective study is required to obtain more insight into the pathophysiology of these diseases.

## Data Availability Statement

All datasets generated for this study are included in the article/supplementary material.

## Ethics Statement

Written informed consent was obtained from the individual(s) for the publication of any potentially identifiable images or data included in this article.

## Author Contributions

CH, PZ, and YL wrote the manuscript. BL and ZH collected the materials. LW and YZ reviewed the whole manuscript.

## Conflict of Interest

The authors declare that the research was conducted in the absence of any commercial or financial relationships that could be construed as a potential conflict of interest.
